# BCDForest: a boosting cascade deep forest model towards the classification of cancer subtypes based on gene expression data

**DOI:** 10.1186/s12859-018-2095-4

**Published:** 2018-04-11

**Authors:** Yang Guo, Shuhui Liu, Zhanhuai Li, Xuequn Shang

**Affiliations:** School of Computer Science and Engineering, Northwestern Polytechnical University, Xi’an, 710072 People’s Republic of China

**Keywords:** Cascade forest, Cancer subtype, Classification

## Abstract

**Background:**

The classification of cancer subtypes is of great importance to cancer disease diagnosis and therapy. Many supervised learning approaches have been applied to cancer subtype classification in the past few years, especially of deep learning based approaches. Recently, the deep forest model has been proposed as an alternative of deep neural networks to learn hyper-representations by using cascade ensemble decision trees. It has been proved that the deep forest model has competitive or even better performance than deep neural networks in some extent. However, the standard deep forest model may face overfitting and ensemble diversity challenges when dealing with small sample size and high-dimensional biology data.

**Results:**

In this paper, we propose a deep learning model, so-called BCDForest, to address cancer subtype classification on small-scale biology datasets, which can be viewed as a modification of the standard deep forest model. The BCDForest distinguishes from the standard deep forest model with the following two main contributions: First, a named multi-class-grained scanning method is proposed to train multiple binary classifiers to encourage diversity of ensemble. Meanwhile, the fitting quality of each classifier is considered in representation learning. Second, we propose a boosting strategy to emphasize more important features in cascade forests, thus to propagate the benefits of discriminative features among cascade layers to improve the classification performance. Systematic comparison experiments on both microarray and RNA-Seq gene expression datasets demonstrate that our method consistently outperforms the state-of-the-art methods in application of cancer subtype classification.

**Conclusions:**

The multi-class-grained scanning and boosting strategy in our model provide an effective solution to ease the overfitting challenge and improve the robustness of deep forest model working on small-scale data. Our model provides a useful approach to the classification of cancer subtypes by using deep learning on high-dimensional and small-scale biology data.

## Background

It is well known that cancer tumor is heterogeneous disease with diverse pathogeneses [1, 2]. Most cancers have multiple subtypes with distinct molecular signatures and likely have different prognoses and treatment responses [3–5]. Recently, the advance of high-throughput profiling technologies has produced huge genomic data and provided unprecedented opportunities to investigate genomic or transcriptomic changes associated with cancers, which makes it possible to cognize cancer subtypes at molecular levels. In the past few years, various types of large-scale genomic data have been used for cancer prognosis integrating gene function studies [6–8] and subtype outcome prediction [1, 4, 9–11], and numerous cancer subtype classification methods have been proposed [5, 12–14]. However, since the complexity of cancer diseases and limited prior knowledge of cancer subtypes [15–17], the overall performance of most current methods still need to be further improved. In general, the intuitive approaches of cancer subtype classification use conventional classification algorithms to learn prediction models based on various types of genomic data and prior subtype knowledge, such as gene expression, DNA-methylation or gene mutations, etc. [18–21]. However, three challenges may limit the application of conventional leaning models, such as SVM, random forest, etc., to the mission of cancer subtype classification on biology data. Firstly, the characteristics of small sample size and high-dimensionality of biology data strengthen the risk of overfitting in training. Secondly, class-imbalance is very common in biology data, which aggravates the difficulties of model learning. Thirdly, large sequencing bias of biology data may weaken the ability of model estimation. Although many modified models have been proposed to ease these challenges in the past few years [5, 22], the alternative options of available methods towards small-scale biology data are still limited, and more accurate and robust methods need to be further developed for the mission of cancer subtype classification.

In recent years, the advance of deep neural networks (DNNs) has achieved great success in various applications, especially in visual and speech recognitions [23, 24]. Inspired by deep neural networks, many methods have been proposed to predict cancer subtypes using variants of deep learning approaches [25, 26]. However, a few deficiencies may limit the applications of deep neural networks in cancer genomic data. On the one hand, deep neural networks are complicated models and huge amount of training data are usually required to train the model [23]. Nevertheless, there aren’t large enough samples for most cancer genomic data at present. On the other hand, it is well known that there are many hyper-parameters in deep neural networks, and the performance of model largely depends on the skills of parameter tuning. This makes it is unruly to get anticipate classification performance using deep neural networks in practice, especially on the small-scale biology datasets.

**Fig. 1:**
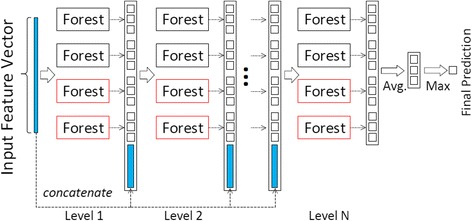
Illustration of cascade forest structure. Each level of the cascade consists of two random forests (black) and two completely random forests (red). Suppose there are three classes to predict; each forest outputs a three-dimensional class vector, which is then concatenated for representation of original input [23]

To ease aforementioned deficiencies of deep neural networks, recently, an alternative to deep learning framework-deep forest model, called gcForest, has been proposed in [23]. Similar to the deep neural network, gcForest has multi-layer cascade structure, but each layer contains many random forests instead of neurons in deep neural networks. Inspired by the deep neural networks, gcForest consists of two ensemble components. The first one is multi-grained scanning, which adopts sliding window structure to scan local context from high-dimensionality to learn representations of input data according to different random forests. The second one is the cascade forest, which learns more discriminative representations under supervision of input representations at each layer, thus gives more accurate predictions according to ensemble of random forests. Unlike deep neural networks, which defines numerous hidden neurons to learn representations layer-by-layer by using forward and backward propagation algorithms, the cascade forest assembles lots of decision tree forests to learn classification distribution (features) according to cascade layers, supervised by input data at each layer. Figure 1 shows a schematic illustration of cascade forest [23]. In cascade forest, each layer of cascade assembles a number of decision tree forests, and receives features processed by its preceding, and inputs its processed results to the next layer. Indeed, each layer is designed to include different types of forests to encourage the diversity of the ensemble. Two types of forests, completely random forest and random forest, are employed in Fig. 1. In each layer, the number of forests and the number of trees in each forest are hyper parameters in practice. An instance is input to a cascade layer, each forest produces an estimate of class distribution. The class distribution outputs by all forests in the same layer form a class vector, and then concatenates with the original vector to be input to the next layer of cascade. In practice, cross validation is used to evaluate the overall performance when a new layer is expanded, and the expanding progress will be automatically terminated once there is no significant performance gain (More details in [23]). As the authors pointed out, the number of cascade layers could be adaptively determined by evaluating the performance at each layer. Therefore, there are fewer hyper-parameters of gcForest than deep neural networks, and it could be trained conveniently without too much parameter tuning skills. However, two challenges of gcForest may limit its application on small-scale biology data. 1). by manually defining different types of forests to encourage the diversity of ensemble in multi-grained scanning, this strategy may raise the risk of overfitting on small-scale or class-imbalance data. 2). all forests in ensemble have equal contributions to final prediction, and the fitting qualities and feature importance aren’t considered in the feature learning process. The final decision of ensemble may be affected by the votes from under-fitting and/or over-fitting forests, especially on small-scale data. In order to take the advantages of gcForest, it is important to modify it to work better on complex biology data.

In this paper, we propose so-called BCDForest (Boosting Cascade Deep Forest) model to follow the mission of cancer subtype classification. The main idea of BCDForest model is to both encourage the diversity of ensemble and consider the fitting quality of each random forest in multi-grained scanning to give more informative presentations of input. We also propose a simple strategy to boost the weights of important features in cascade random forests, thus to improve the overall performance of cascade ensemble random forests. Our contributions can be summarized as follows. 1). we adopt a multi-class-grained scanning strategy to encourage the diversity of ensemble by using different training data of classes respectively. Different training sub-datasets are used to construct various types of forests to encourage the diversity of ensemble. 2). we consider the model fitting qualities of forests in feature representation learning by using sliding window scanning. The out-of-bagging [27, 28] approach is used to estimate the error of model fitting and assign a confidence weight to each forest to correct the outcome predictions. 3). we propose a variation based strategy to boost important features in forest learning at each layer of cascade forest.

Applying BCDForest to three public microarray gene expression datasets and six RNA-Seq gene expression datasets from TCGA [29, 30], we find that BCDForest has better prediction performance than conventional methods and gcForest. Importantly, BCDForest achieves higher prediction accuracy than the standard gcForest on small-scale (small sample size) and class-imbalance datasets, which is crucial to the supervised learning of cancer genomic data.

## Methods

### Boosting cascade forest

The cascade forest model provides an alternative to deep neural networks (DNNs) to learn hyper-level representations in low expense. Instead of learning hidden variables according to complex forward and backward propagation algorithms in DNNs, cascade forest tries to learn class distribution features directly by assembling amounts of decision tree-based forests under supervision of input. The layer-wise supervised learning strategy allows cascade forest can be easily trained. In addition, the ensemble of forests hopes to obtain more precise class distribution features, as it is well known that the random forest has powerful classification ability in most applications. However, in the standard deep cascade forest model, the feature importance isn’t considered in representation learning process among multiple layers. This may lead to the overall prediction performance is sensitive to the quantities of decision trees in each forest, especially on small-scale data, since it is crucial to select the discriminative features as splitting nodes to construct decision trees. Based on the basic architecture of cascade forest, in this section, we introduce a novel modified version of cascade forest, which is denoted as BCDForest.

Inspired by the boosting idea, we assign the discriminative features with higher weights than uninformative features in forest training process. Obviously, it is hard to give a meaningful weight to each feature directly in the output concatenated vector at each cascade layer, as it is a combinatorial class distribution of global and local features. On the one hand, different random forests may offer different estimations, we don’t have any weight information about their estimations. On the other hand, extensive weight estimations of features will introduce additional expense. In this study, alternatively, we try to emphasize the discriminative features in the concatenated vector to boost the possibility of these features to be selected as splitting features of decision trees in each random forest, thus to encourage generating better fitting forests. In fact, the importance of each feature can be predicted by the structures of decision trees in their fit random forest. In detail, the features in high level of decision trees tend to be more important to discriminate different classes on training data, so they should be boosted as more important features that need to be reconsidered in the next layer. Given a fit forest, by combining all decision trees, the importance of features can be easily predicted by considering the average height rank of features in all decision trees of forest. We select the top-*k* most important features in each forest, and use the standard deviation of the *k* features to compose a new feature. Then, we combine the new variance feature with the output class distribution vector together to boost its class distribution in the concatenated input vector of next layer, thus to reduce the false discovery rate of estimation in the next propagation layer. The reasons for using the standard deviation of top-*k* features instead of using top-*k* features directly are: 1). to reduce the sensitivity of model to the *k* parameter; 2). the variance can present the difference of instances on top-*k* features in some extent. This boosting operation can be implemented at each layer of cascade forest, and it doesn’t introduce additional computational expense, since the importance of features can be easily estimated by generating forests.

**Fig. 2:**
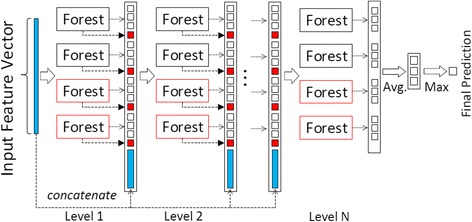
Illustration of boosting cascade forest structure. Each level of the cascade consists of two random forests (black) and two completely random forests (red). The standard deviation of top-*k* important features in each forest will compose a new feature to be concatenated in the next cascade level to emphasize the discriminative features

Figure 2 illustrates the basic architecture of our boosting cascade forest model. Given an instance, each forest produces an estimate of class distribution as described in [23]. Meanwhile, we select top-*k* features from each fit forest and construct the standard deviation feature on each forest, and then concatenate all new features with their corresponding output class distribution vectors to generate boost class distribution vectors. Finally, we concatenate all of boost estimate vectors of forests at each layer with the original feature vector as the input of the next layer of cascade. In detail, the same as the standard cascade forest model, we set up two completely random forests (using all features, gini value based) and two random forests at each layer of cascade, and 1000 decision trees in each forest. We define the default value of boosting parameter *k* = 5. To ease the risk of the overfitting, we use cross-validation method to evaluate the overall performance at each layer, and the propagation of cascade will be automatically terminated once the performance turns to decrease. At last, the final fitting cascade forest model will be used to estimate class labels of new instances.

### Multi-class-grained scanning

Inspired by deep neural network in handling feature relationships, cascade forest employs multi-grained scanning strategy, a sliding window based approach, to extract local features by scanning raw input to generate a series of local low-dimensional feature vectors, and then train a series of forests by using those low-dimensional vectors to obtain class distributions of input vectors (more details in [23]). Although it has been proved the multi-grained scanning is effective on local feature recognition, a few drawbacks may affect the quality of the extracted features in applications. 1). to consider a class-imbalance data, the decision trees of forests tend to underline the classes with most instances, and block the recognition of small-size classes in training, especially on the high-dimensional data. 2). the diversity of forests depends on manual hard-definition, not automatically determines in data-driven way. This may weaken the ability of classification, as the diversity is crucial to ensemble construction [23], especially on small-scale data. 3). all scanning forests in ensemble have equal contributions in multi-grained scanning, and it may lead to the estimation of classification distribution is sensitive to the fitting quantities of forests. To ease these issues, in this study, we propose a multi-class-grained scanning approach to encourage the diversity of ensemble forests by using different training data of classes.

**Fig. 3:**
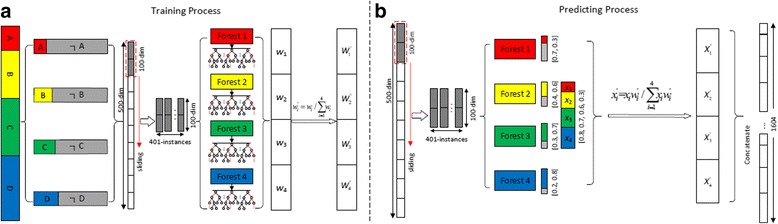
Illustration of multi-class-grained scanning. **a** Suppose four classes (A, B, C and D) in training dataset. For each class, we produce the positive and negative sub-datasets, and then use the sub-datasets to train a binary random forest classifier. Four different types of random forests will be produced by using different training datasets (sliding window based). The out-of-bagging (OOB) score of each forest is used to calculate a normalized quantity weight to each forest. **b** Based on the fit forests and their quantity weights, a 500-dim instance vector can be transformed to a concatenated 1604-dim representation

As shown in Fig. 3a, suppose there are *m* instances from 4 classes in training data. We first simulate multiple sub-datasets according to different class labels. In detail, for each class, we produce a sub-dataset, which consists of positive and negative instances respectively. For example, assume there are *n*_*A*_ instances in class *c*_*A*_, the instances in class *c*_*A*_ are the positive set, and the non *c*_*A*_ instances are the negative set. Therefore, in each sub-dataset, only two types of instances are denoted. At last, 4 sub-datasets are produced, and each sub-dataset is used to train a forest to sense a specific class. Similarly, for each instance in sub-dataset *c*_*i*_, assume there are 500 raw features and the slide window size is 100, then 401 feature vectors are produced by sliding the window for one feature each time. All feature vectors extracted from positive/negative raw instances are regarded as positive/negative instances. The instances from the same sub-dataset will be used to train a random forest, and 4 forests will be produced for all 4 sub-datasets. For each raw instance, all of sliding feature vectors are input to all of 4 forests and generate their class distribution vectors, and then concatenate them as transformed features. As shown in Fig. 3b, 100-dimensional window size is used, and 401 feature vectors will be extracted from each raw instance. For each 100-dimensional vector, each forest generates a 2-dimensional class vector, and 4 class vectors are generated in total. We select the possibility of positive estimation in each of 2-dimensional class vector and concatenate them to generate a 4-dimensional transformed class vector. Importantly, since the difference of training data, such as sample size, etc., may affect the quality of model, it is important to consider the fitting qualities of scanning forests when predicting the class distribution of input instances. We use the out-of-bagging (OOB) [27, 28] method to measure fitting quantity of each scanning forest, thus produce a quantity weight to each scanning forest.

Formally, we normalize each 4-dimensional vector into a class distribution space. Suppose *X* = (*x*_1_, *x*_2_, *x*_3_, *x*_4_) is a 4-dimensional class vector, *W* = (*w*_1_, *w*_2_, *w*_3_, *w*_4_) is the vector of out-of-bagging fitting score for all scanning forests, ${W}^{\prime }=\left({w}_1^{\prime },{w}_2^{\prime },{w}_3^{\prime },{w}_4^{\prime}\right)$, where ${w}_i^{\prime }={w}_i/\sum \limits_{i=1}^4{w}_i$, is the weight vector of forests, and the normalized class vector is defined as ${X}^{\prime }=\left({x}_1^{\prime },{x}_2^{\prime },{x}_3^{\prime },{x}_4^{\prime}\right)$, where ${x}_i^{\prime }={x}_i{w}_i^{\prime }/\sum \limits_{i=1}^4{x}_i{w}_i^{\prime }$. Each 100-dimensional vector is transformed into a 4-dimensional normalized class vector, and all of 401 4-dimensional class vectors are concatenated to a 401 × 4-dimensional class vector, corresponding to the original 500-dimensional raw feature vector. Figure 3 shows only one window size, but multiple window sizes could be defined by user to result in more features in the final transformed vectors.

In addition, if there are many classes in the training data, we still can divide the raw data into different class groups by the principle of leaving label balance, and thus simulate multiple sub-datasets, and train multiple types of forests. Note, the most difference between our multi-class-grained scanning and the standard multi-grained scanning is that we use different sub-datasets to train different forests to encourage the diversity of ensemble. It is a data-driven way to train different forests, thus to extract more meaningful local features from raw features. The three most advantages of our approach can be summarized as: 1). dividing training data into different sub-datasets can ease under estimation caused by class-imbalance data. 2). the number of forests is determined by the classes in raw data instead of a hyper-parameter to ease the risk of overfitting. 3). it encourages the diversity of forests and heartens to give more accurate classification by assembling more simple classifiers.

### Overall procedure of BCDForest

**Fig. 4:**
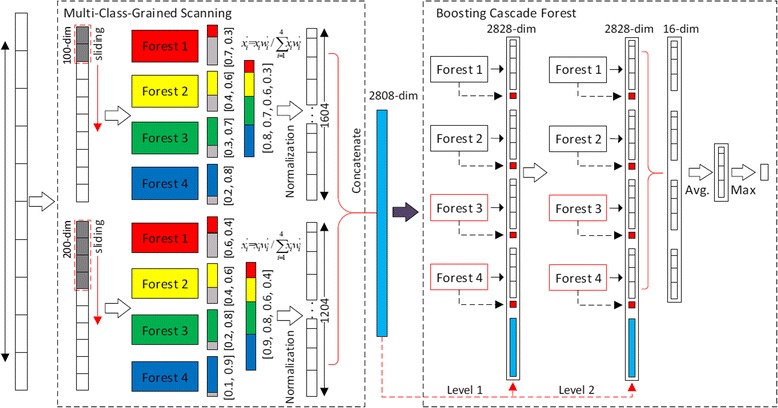
Overall procedure of BCDForest. Suppose there are four classes, and the sliding windows are 100-dim and 200-dim. Two cascade layers are used to give final prediction

The general BCDForest framework includes two main components. The first one is the multi-class-grained scanning, which learns the local-context based on class distribution representations of input data according to different forests. The second one is the boosting cascade forest structure, which considers feature importance in cascade layers and learns more discriminative representations under supervision of input at each layer. Figure 4 illustrates an example of overall procedure of BCDForest. Suppose the input is of 500-dimensional data, and two window sizes (100, 200) are used in multi-class-scanning. There are 4 classes and *m* instances in training data. 4 sub-datasets are simulated based on the positive and negative instances of each class, and 4 forests (each contains 50 trees) can be learned from all sub-datasets. The window size of 100 features generates 401 × 100-dimensional feature vectors for each raw instance in training data, and each 100-dimensional vector is transformed to a 4-dimensional class vector. Then a 1604-dimensional transformed feature vector is obtained at last. Similarly, the sliding window with 200 size generates a 1204-dimensional feature vector for each original instance. By concatenating those two vectors, a 2808-dimensional feature vector is produced, which is a representation of the original 500-dimensional feature vector. All *m* 500-dimensional original vectors are presented by *m* 2808-dimensional feature vectors, and then they are input to the boosting cascade forests. Suppose top-k features are selected to extract the standard deviation boosting feature in each forest, and 4 forests are assembled at each layer of cascade, then for each raw 500-dimesional feature vector, a 2828-dimensional feature vector is output at the first layer of cascade. All these 2828-dimensional feature vectors are input to the next layer of cascade as training data; and this process is repeated until the validation performance suggests that the expanding of cascade should be terminated. Finally, given a test instance, it is transformed into 2808-dimensional representation vector at first by using multi-class-grained scanning. Then, the representation is input into the boosting cascade forest structure to get its final prediction by aggregating the four 4-dimensional vectors at the last layer of cascade, and taking its class with the maximum aggregated value.

## Results

### Datasets and parameters

To investigate the effectiveness of BCDForest, we conducted cancer subtype predictions on both microarray and RNA-Seq gene expression datasets. We downloaded three microarray gene expression datasets of adenocarcinoma, brain and colon cancer types respectively [31]. The detail of these three datasets are shown in Table 1. We also downloaded RNA-Seq gene expression data of five cancer types (BRCA (breast invasive carcinoma), GBM (glioblastoma multiforme), LUNG (lung cancer), COAD (colon adenocarcinoma) and LIHC (liver hepatocellular carcinoma)) and integration of pan-cancers (includes 11 cancers) data from TCGA [29, 30]. We selected these cancer data because these cancer types were well studied over the past decade years. For the integration of pan-cancers data, we filtered out the genes having missing values in samples, and logarithm transformed data to normalized values were used in downstream analyses. For the five cancer types data, the gene expression were logarithm transformed data, and we selected the informative genes as transcriptome features in classification analyses by two categories: 1) average gene expression> 5.0; 2) variance of gene expression among samples > 1.0. The details of the datasets are shown in Table 2. For each dataset, we downloaded the corresponding clinical data from TCGA, and used the clinical subtype information to label each sample, thus to be used to evaluate the performance of our method. In all experiments of this study, we set 50 decision trees in each forest of multi-class-grained scanning, and 1000 decision trees in each forest of boosting cascade forest at default. The completely random forest is generated based on gini values using all input features in gcForest and BCDForest. Two completely random forests and two random forests were set in each layer of boosting cascade forest, and the top-5 most important features were considered.

**Table 1:** Comparison of overall accuracy on microarray gene expression datasets

Code	Dataset	Sample	Gene	Class	Overall Accuracy					
					KNN	LR	RF	SVM	gcForest	BCDForest
1	Adenocarcinoma	76	9868	2	0.842	0.736	0.841	0.842	0.857	0.928
2	Brain	42	5597	5	0.784	0.858	0.796	0.690	0.892	0.964
3	Colon	62	2000	2	0.801	0.660	0.846	0.885	0.916	0.916

**Table 2:** Comparison of overall accuracy on RNA-Seq gene expression datasets

Code	Dataset	Sample	Gene	Class	Overall Accuracy					
					KNN	LR	RF	SVM	gcForest	BCDForest
1	PANCANCER	3594	8026	11	0.955	0.979	0.960	0.968	0.965	0.973
2	BRCA	514	3641	4	0.778	0.854	0.845	0.793	0.881	0.920
3	GBM	164	3180	4	0.694	0.651	0.702	0.619	0.741	0.806
4	LUNG	275	4000	3	0.710	0.744	0.791	0.786	0.830	0.867
5	COAD_I	264	3010	6	0.348	0.287	0.377	0.372	0.392	0.411
6	COAD_N	270	3006	3	0.699	0.631	0.696	0.700	0.711	0.730
7	COAD_T	282	3014	3	0.766	0.701	0.767	0.765	0.767	0.785
8	LIHC_I	347	4401	3	0.532	0.491	0.536	0.527	0.558	0.588
9	LIHC_N	400	4398	2	0.695	0.519	0.698	0.696	0.708	0.759
10	LIHC_T	347	4347	3	0.574	0.503	0.579	0.561	0.608	0.652

### Overall performance on microarray datasets

We compared the classification performance of BCDForest with four conventional methods (KNN, SVM, Logistic Regression (LR) and Random Forest (RF)) and the standard gcForest on three microarray datasets. To perform the classification estimate, the most challenge of those three datasets is the data characteristics of small sample size but high-dimensional gene features. We used 5-fold cross validation to evaluate the overall accuracy of different methods on these three datasets. In fair, in each class of datasets, we randomly selected 4/5 samples for training data, and 1/5 samples for testing data. As shown in Table 1., BCDForest consistently outperforms other methods in overall accuracy prediction. This illustrates that our method is effective to cancer subtype classification on small-scale data. This may be for the reason that BCDForest uses simple binary forests to learn the classification distribution features in multi-class-grained scanning, and it can ease the risk of overfitting in some extent. In addition, we find both gcForest and BCDForest outperform other conventional methods on these three small-scale datasets, especially on the adenocarcinoma dataset, which includes 9868 genes, but only 76 samples, both gcForest (85.7%) and BCDForest (92.8%) obtain higher accuracy. This demonstrates that the deep forest framework has powerful ability to cancer subtype classification than others on small-scale microarray datasets.

### Overall performance on RNA-Seq datasets

To systematically investigate the robustness of BCDForest, we examined the estimate performance on more datasets, comparing with four conventional methods and the standard gcForest. In order to test the performance of BCDForest on relatively large-scale dataset, we downloaded the integrated pan-cancers dataset from TCGA, which included 3594 samples from 11 different cancer types, and the class sample size ranged from 72 to 840 respectively. We used the tumor cancer type to label each sample, and trained each model based on these labels. We used 5-fold cross validation to evaluate the performance of each method on these datasets. In fair, in each class, we randomly selected 4/5 samples to compose the training data, and 1/5 samples to compose the testing data. Table 2. shows the overall accuracy performance of different methods on integrated pan-cancer dataset. We also downloaded gene expression and clinical data of other five cancer types (BRCA, GBM, LUNG, COAD and LIHC) from TCGA. Specifically, the BRCA, GBM and LUNG datasets have cancer subtype label information in the clinical data. We used their subtype labels directly in experiments. For the COAD and LIHC datasets, there wasn’t known subtype information in the clinical data, while the pathologic states were depicted. We used three of clinical pathologic states (pathologic stage (I), pathologic N and pathologic T) to define three different cancer sub-datasets, which stated different pathologic class labels. In particular, we filtered out the pathologic subtypes with few samples in each data to reduce the effect of outliers. Table 2. shows the details of each dataset and the overall performance of each method on each dataset.

As shown in Table 2., on the large-scale pan-cancers dataset, all methods have similar prediction performance, although the LR (97.9%) and BCDForest method (97.3%) have slightly higher accuracy than others. The reason may be that there are different gene expression patterns among different cancer types. However, on the other small-scale cancer datasets, BCDForest is consistently better than other methods, especially comparing with the conventional methods. For example, on the GBM data, BCDForest method obtains the highest accuracy (80.6%), and it is better than gcForest (74.1%) over 6.5%, and better than RF (70.2%) over 10%. In addition, it is interesting that both BCDForest and gcForest are better than other conventional methods on all five cancer types of datasets. This indeed demonstrates that the deep forest methods are more powerful to the classification of cancer subtypes since more complex features can be learned to discriminate different classes.

### Cancer type classification on pan-cancers dataset

**Fig. 5:**
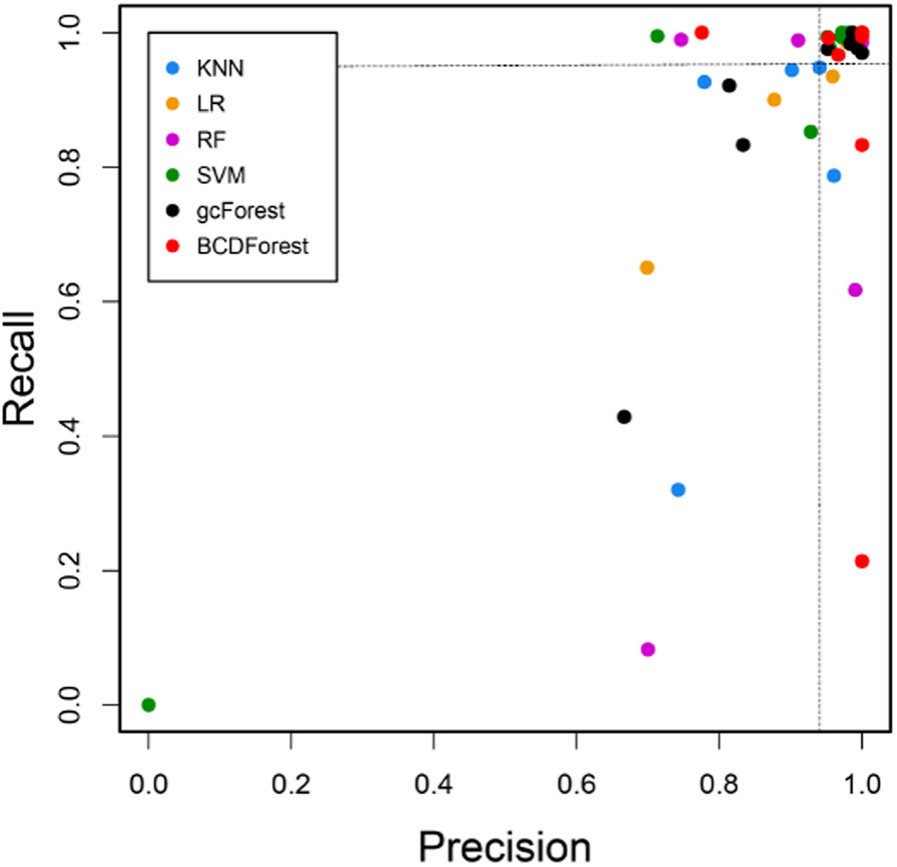
Comparison of different methods on large-scale pan-cancers dataset. Each dot presents the performance of each corresponding method on each cancer type. 11 cancer types were included in the pan-cancers dataset

To examine the robustness of BCDForest on different class sample sizes, we conducted experiment of cancer type classification on TCGA pan-cancers dataset. For each cancer type, we randomly selected 4/5 samples for training, and 1/5 samples for testing. In total, 2875 samples derived from all of 11 cancer types were used for training, and 719 samples derived from all of 11 cancer types were used for testing. Then, we investigated the *precision* and *recall* performance of each method on each cancer type data. As shown in Fig. 5, our BCDForest method has the best *precision* and *recall* performance on most of cancer types comparing with other methods, although most of other methods also have good performance. In addition, BCDForest tends to give higher *precision* performance than other methods on most of cancer subtypes (as the dash line shown). This illustrates BCDForest is more robust to various cancer types since it could sense more information in training data.

### Cancer subtype classification on BRCA, GBM and LUNG datasets

**Fig. 6:**
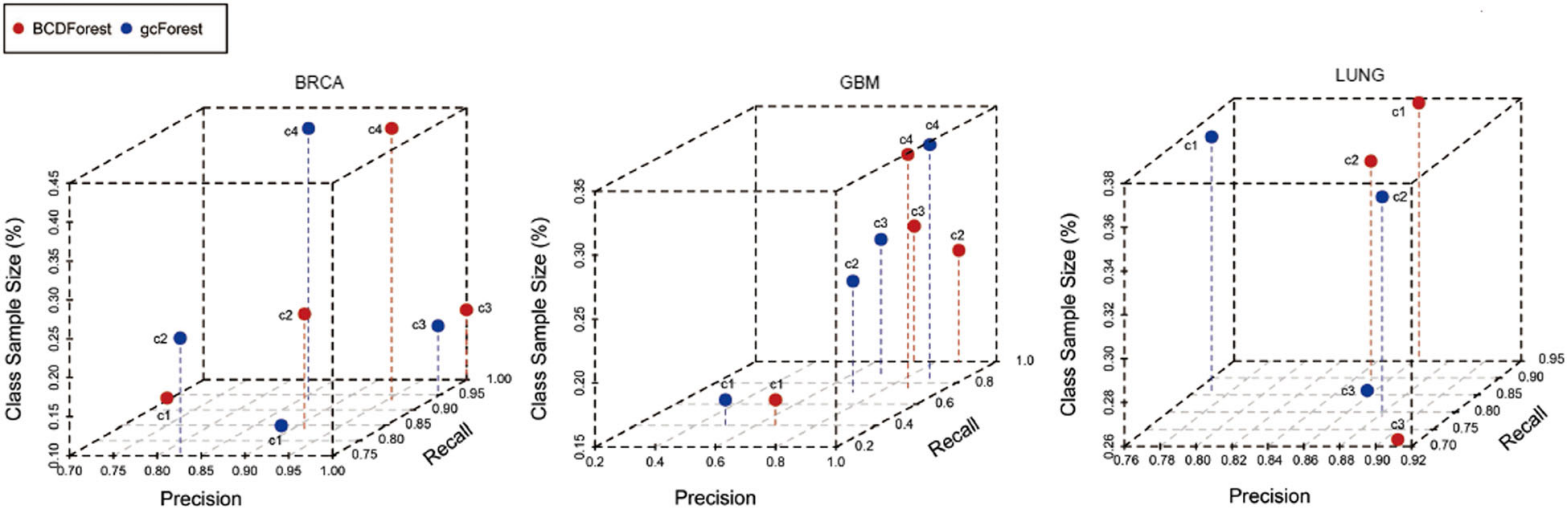
Comparison of BCDForest and gcForest on three cancer type datasets (BRCA, GBM and LUNG). Each dot presents the performance of each method on each cancer subtype class

**Fig. 7:**
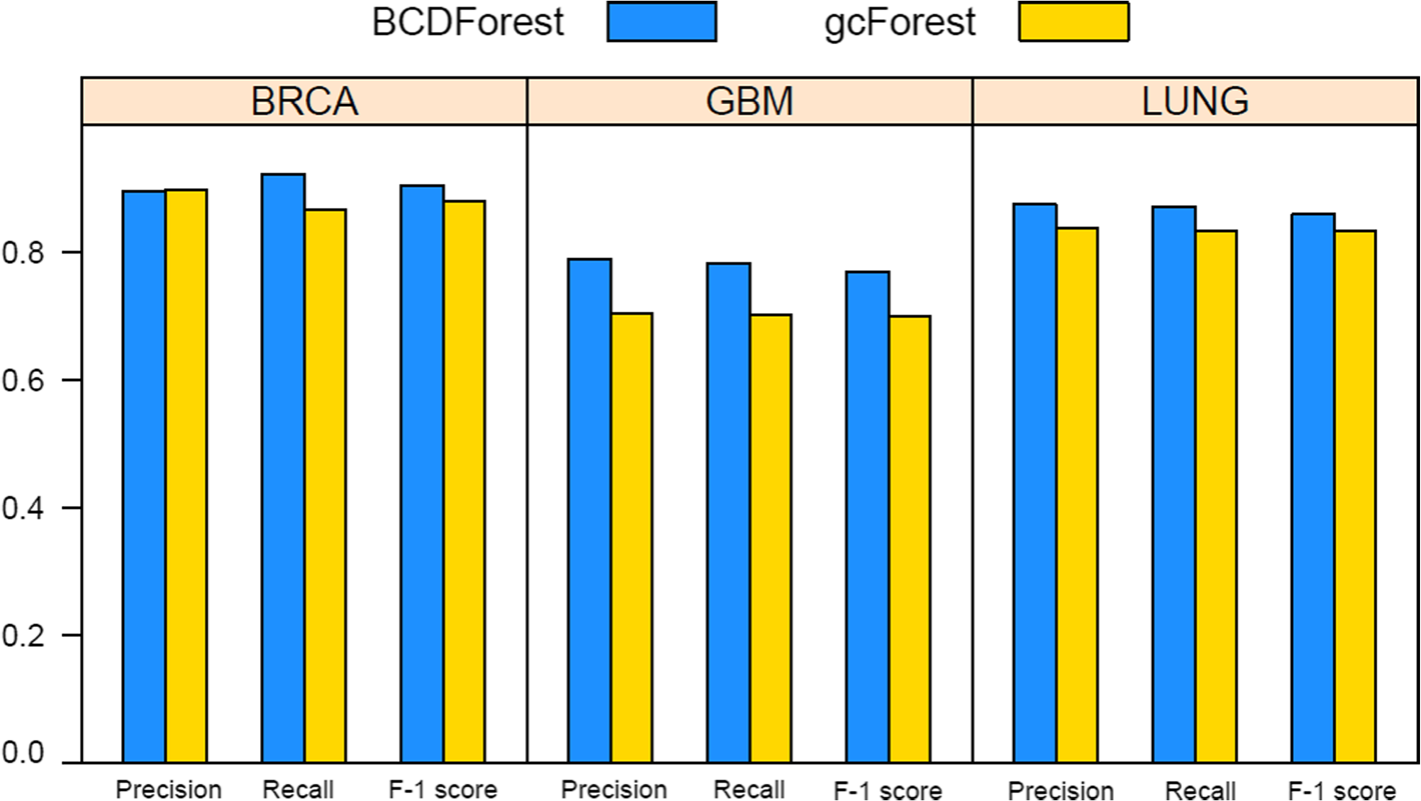
Comparison of overall performance of BCDForest and gcForest on BRCA, GBM and LUNG datasets. The average *precision*, *recall* and *F-1* score on all subtype classes of each dataset were evaluated

Since BCDForest was proposed based on the gcForest, we conducted two experiments to examine the performance of BCDForest and the standard gcForest on three cancer datasets (BRCA, GBM and LUNG), respectively. It is well known that the class balance of training data is very important for model learning. However, the situation of class-imbalance is very common in many fields, especially in biology data, and it can affect the performance of model learning and prediction [32]. To evaluate the robustness of our method to class-imbalance data, we investigated the sensitivity of the two deep forest methods to proportion of sample size on each of three datasets. Then, we evaluated the average *precision*, *recall* and *F-1* score performance of each method in all classes of each dataset. We selected BRCA, GBM and LUNG cancer datasets because these datasets included cancer subtype information in clinical data. Specifically, in BRCA data, there are four basic subtypes in 514 samples (Basal-like: 98/~ 19.06%, HER2-enriched: 58/~ 11.28%, Luminal-A: 231/~ 44.94%, Luminal-B: 127/~ 24.71%); in GBM data, there are four basic subtypes in 164 samples (Classical: 42/~ 25.61%, Mesenchymal: 55/~ 33.5%, Neural: 28/~ 17.07%, Proneural: 39/~ 23.78%); in LUNG data, there are three basic subtypes in 275 samples (Bronchioid: 104/~ 37.81%, Magnoid: 72/~ 26.18%, Squamoid: 99/~ 36.0%). In each cancer subtype, we randomly selected 4/5 samples for training, and 1/5 samples for testing. As shown in Fig. 6, comparing with gcForest, BCDForest has better performance for both *precision* and *recall* on most of subtype classes of three datasets. Importantly, BCDForest seems to be more robust to the class-imbalance data. For example, even on the smallest subtype class in GBM (c1, ~ 17.07%), BCDForest has higher *precision* and *recall* prediction comparing with gcForest. On the BRAC data, BCDForest has higher *precision* and *recall* performance on three out of four subtype classes comparing with gcForest. Particularly, on the smallest subtype class (c1, ~ 11.28%), although the *precision* performance of BCDForest is not better than gcForest, it has higher *recall* performance than gcForest. This illustrates that BCDForest tends to be more sensitive to the positive samples in class-imbalance cases. To evaluate the overall performance of the two deep forest approaches on the three datasets, we examined the average *precision*, *recall* and *F-1* score performance of each method in all classes of each dataset. As shown in Fig. 7, BCDForest consistently outperforms the standard gcForest in cancer subtype prediction on all three datasets, especially on GBM cancer data (the average *F-1* score higher than gcForest over 7.0%). This demonstrates that BCDForest is more robust than the standard gcForest to discriminate the cancer subtypes on small-scale cancer datasets, since it considers more feature information in estimation.

### Pathologic cancer subtype classification on COAD and LIHC datasets

**Fig. 8:**
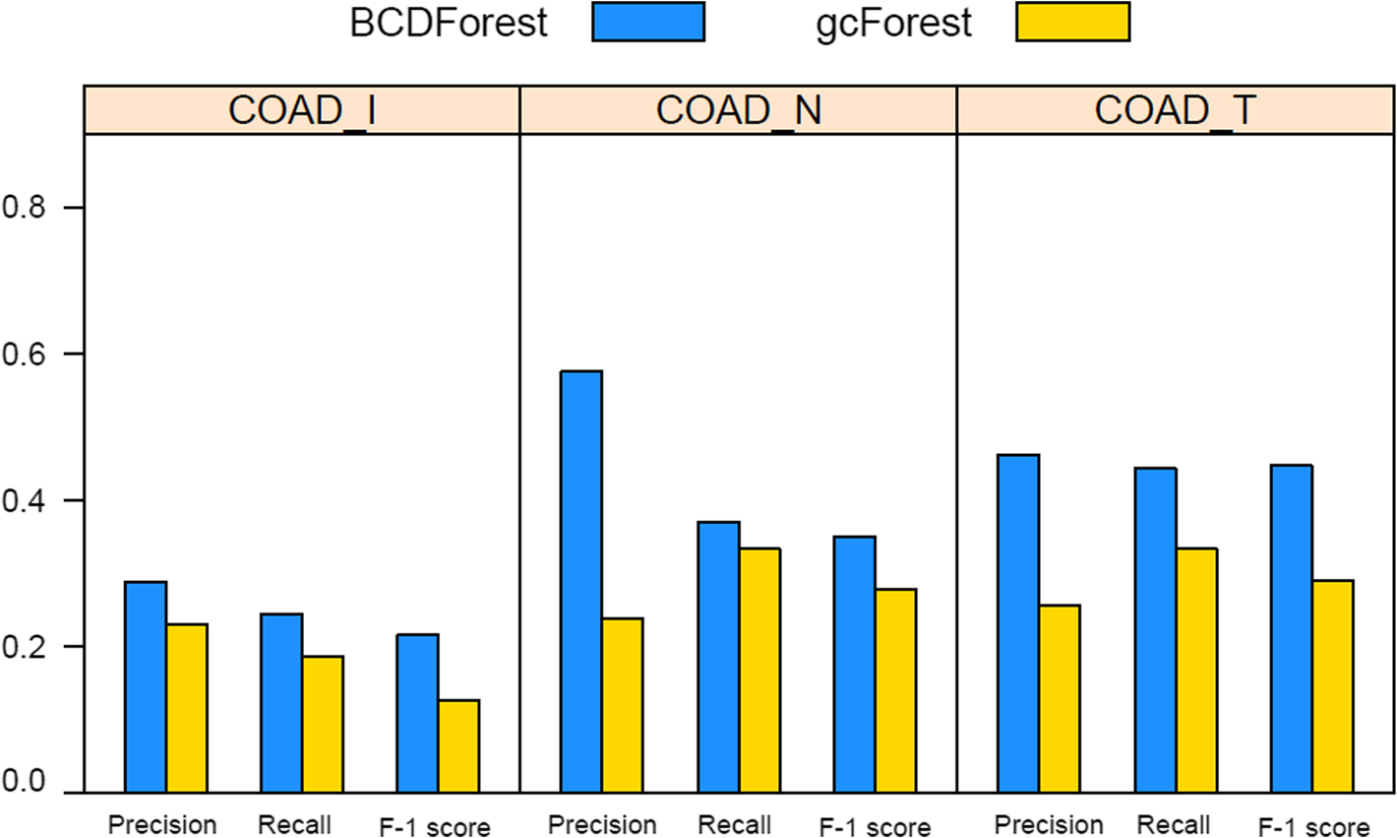
Comparison of overall performance of BCDForest and gcForest on COAD datasets. The average *precision*, *recall* and *F-1* score on all subtype classes of each dataset were evaluated

**Fig. 9:**
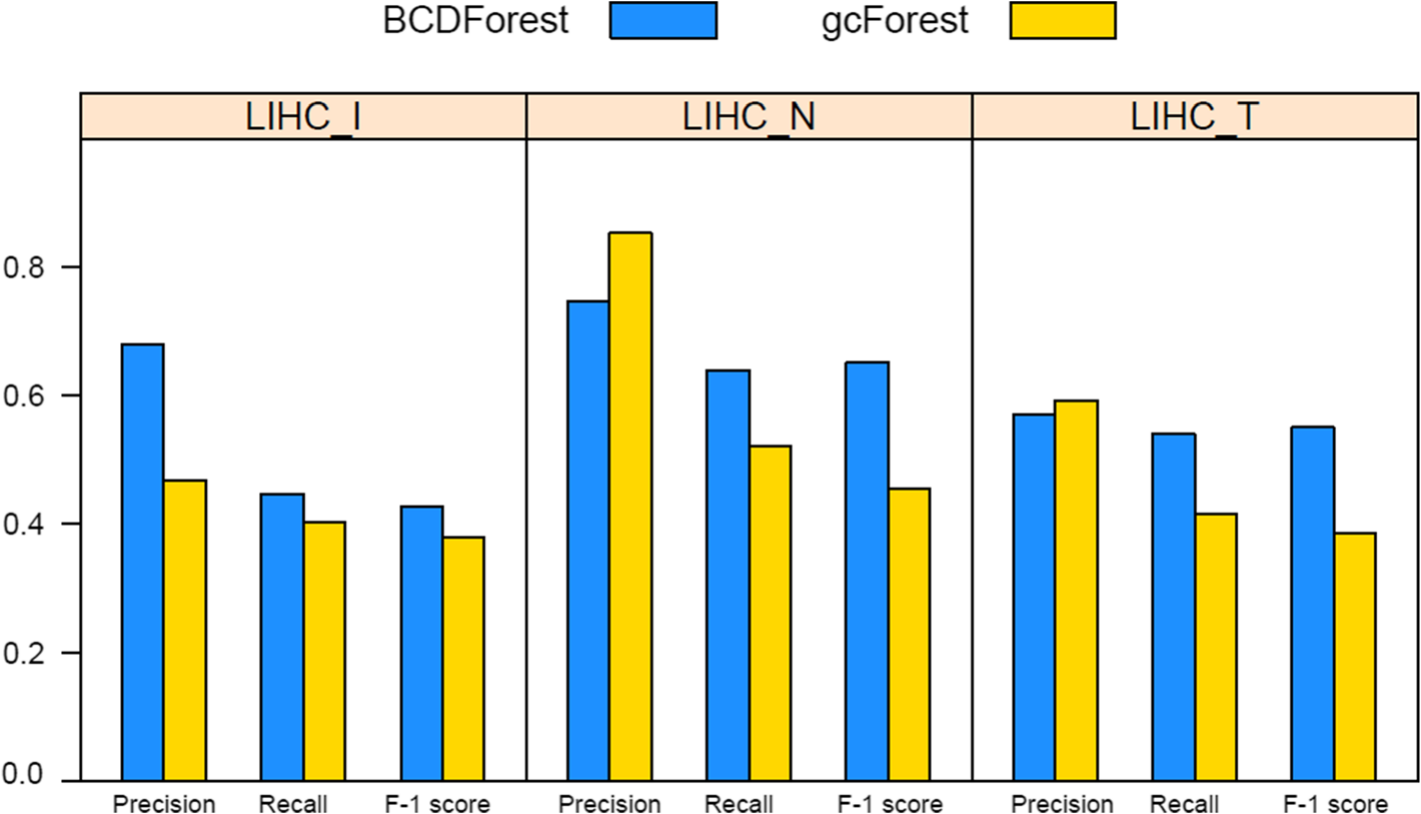
Comparison of overall performance of BCDForest and gcForest on LIHC datasets. The average *precision*, *recall* and *F-1* score on all subtype classes of each dataset were evaluated

The different pathologic states of cancer tumors could be used to define pathologic meaning subtypes in some extent. To systematically investigate the robustness of our method in discriminating of cancer subtypes, we also examined the performance of BCDForest and the standard gcForest for pathologic subtype prediction on COAD and LIHC datasets. For the COAD and LIHC datasets, since there were not specific cancer subtype information in the clinical data, we used different types of pathologic states of patients to define different pathologic subtypes on each dataset. We used the same method (4/5 samples for training; 1/5 samples for testing) to evaluate the overall performance of BCDForest and gcForest respectively on each dataset. Specifically, we evaluated the average *precision*, *recall* and *F-1* score in all classes on each dataset. For these two datasets, we used three different types of pathologic states (pathologic N, pathologic T, pathologic stage (I)) to define different subtype labels of patients, thus to build different classification missions on six sub-datasets. In detail, we filtered out the subtype classes which had too few samples in all of datasets. At last, we performed classification on all six sub-datasets which defined by different types of pathologic states on the two cancer types. Specifically, for COAD data, three sub-datasets were generated based on different types of pathologic stages (pathologic_N (N0, N1, N2), pathologic_T (T2, T3, T4), pathologic_I (I, II, IIA, IIIB, IIIC, IV)); for LIHC data, three sub-datasets were generated based on different types of pathologic stages (pathologic_N (N0, NX), pathologic_T (T1, T2, T3), pathologic_I (I, II, IIIA)). The details of each sub-dataset are shown in Table 2. As shown in Fig. 8, BCDForest consistently outperforms gcForest on three sub-datasets of COAD. Especially, on the pathologic_I and pathologic_T sub-datasets, BCDForest has significantly higher *F-1* score than gcForest. This illustrates our method is more robust on small-scale datasets. As shown in Fig. 9, on the three LIHC sub-datasets, we see similar performance except on the sub-dataset of pathologic_N. On the sub-dataset of LIHC_N, although the average *precision* of our method is not as good as gcForest, the average *recall* and *F-1* score are better than gcForest. This indeed demonstrates that our method is more sensitive to the positive samples. In conclusion, based on systematical evaluations on six of pathologic sub-datasets, our BCDForest is more effective and robust than gcForest working on small-scale cancer datasets.

## Discussion

The deep forest framework provides an alternative to deep learning in practice. The standard deep forest model may face overfitting and ensemble diversity challenges working on small-scale biology data. BCDForest is a novel modification of the standard deep forest model (gcForest), and it provides an effective solution to ease the overfitting challenge and improves the robustness of the standard deep forest model working on small-scale biology data. We compared BCDForest with the standard gcForest and several conventional classification methods on both microarray and RNA-Seq gene expression cancer datasets. We found: 1). the deep forest methods (BCDForest and gcForest) consistently outperformed other conventional classification methods on most of cancer datasets. This may be because the deep forest methods can learn more meaningful high-level features in supervised learning. 2). BCDForest consistently outperformed the standard gcForest on most of cancer datasets. This illustrates that our boosting strategies are effectively to improve the classifying ability of the standard deep forest model on small-scale biology cancer datasets, and it provides a robust model to the classification of cancer subtypes. In addition, although our BCDForest model tends to give better prediction performance than the state-of-the-art methods in cancer subtype predictions, some challenges still need to be further fixed, such as working on extremely class-imbalance and high-dimensionality small-scale datasets and improving the stability further, etc. Besides, it has been proved that integrating multiple types of genomic data can benefit the performance of cancer subtype prediction in recent years [33–35]. In this study, we only focused on cancer subtype classifications based on gene expression data. It will be useful to extend the deep forest model to integrate multiple types of genomic data to advance the performance of cancer subtype classification.

## Conclusions

The classification of cancer subtypes is vital to cancer diagnosis and therapy. In this paper, we proposed a deep learning model, so-called BCDForest, to address cancer subtype classification on small-scale biology data, which can be viewed as a modification of the standard gcForest presented recently in [23]. On the one hand, instead of manually defining different types of complex random forests, we proposed a named multi-class-grained scanning strategy to encourage the diversity of ensemble by training multiple simple binary classifiers using the whole training data. Meanwhile, we considered the fitting quality of each simple classifier in representation learning to encourage the accuracy of estimations. On the other hand, we proposed a boosting strategy to emphasize the important features in cascade forests, thus to propagate the benefits of discriminative features among cascade layers. Systematic comparison experiments on both microarray and RNA-Seq gene expression datasets demonstrate that our method consistently outperforms the state-of-the-art methods. In conclusion, our BCDForest method provides an option to investigate cancer subtypes by using deep learning on small-scale biology datasets.
